# Immunity with Reference to Some of Its Relations to Surgery

**Published:** 1912-09

**Authors:** Ludwig Hektoen

**Affiliations:** Director of the Memorial Institute for Infectious Diseases; Chicago


### BUFFALO MEDICAL JOURNAL

Vol. LXVIII SEPTEMBER, 1912 No. 2

### ORIGINAL ARTICLES

### Immunity

**With Reference to Some of Its Relations to Surgery**

Being the Harrington Lectures before the Alumni Association of the University of Buffalo, May 29 and 30, 1912.

By LUDWIG HEKTOEN, M. D.
Director of the Memorial Institute for Infectious Diseases
Chicago

I.

IN speaking of immunity we have in mind primarily the general conditions and processes of the body that enable it to resist infection more or less completely. The study of immunity includes the study of the mechanisms whereby the body protects itself from infection and recovers from infection. As an outcome of such study we have learned to employ some of these natural means with success and we are constantly striving to extend the use of natural means for the purposes indicated and if possible to improve on them; the study of immunity, that is, of the complex responses of the body to infection, has resulted also in giving us new and valuable means of diagnosis.

The underlying principles of immunity are of course the same no matter what the particular point of view from which its manifold phenomena or its practical application may be considered. On this occasion it is my purpose, first, to outline briefly some of the fundamental facts in regard to immunity and, secondly, to illustrate their bearing on treatment and diagnosis by reference to conditions that are perhaps of special interest to the surgeon.

The history of the new science of immunology is a most interesting one; it is especially instructive to trace the early foreshadowings of the great discoveries in this field in recent times, but the time at our disposal does not permit me to recount in a leisurely manner the various steps in the development of our knowledge of immunity from the remote period when Eastern people first practiced inoculation of small-pox to the present day with its serum therapy, its protective and curative inoculations, and its diagnostic serum reactions.

### Antigens and Antibodies.

Preventive and curative inoculation, serum diagnosis, and serum treatment are all based on the wonderful power of the animal body under suitable circumstances to produce substances that neutralize toxic microbic products, destroy the microbes, and break up foreign protein substances. The specific substances, which are concerned in these actions and which manifest themselves in various ways, are now commonly called antibodies, and in the language of the immunologist, any substance that can incite the formation of antibodies is an antigen. It has been found that antigens are protein substances, and that practically any protein that is foreign to an animal will exercise antigenic effects if it is introduced into its blood or tissues. Such proteins may be of vegetable, of bacterial or of animal origin. The red corpuscles and other cells, and the serum as well as other protein-containing substances of animals, all constitute or contain antigens and on that account are used extensively in the investigation of immunological problems; but from the point of view of the practitioner and the patient the most important antigenic substances are furnished by the microbes and the microbic products that are concerned in infectious processes.

### The Actions of Antibodies: Antitoxic Immunity.

Let us now consider briefly some of the chief facts about antibodies, their modes of action, and the laws governing their formation.

The first antibody with which we were made familiar is diphtheria antitoxin which was discovered by Behring in 1890. This great discovery was made possible by the discovery shortly before, by Roux and Yersin, that the diphtheria bacillus secretes a toxin which causes the essential disturbances of diphtheria infection. Behring found that certain animals respond to the injection of increasing quantities of toxin by the production of a specific antitoxin in far greater amounts than necessary for their own protection, and furthermore, that the serum of animals immunized in this way, when injected into other animals, may protect them from the corresponding toxin and even cure such as are suffering from the effects of the toxin. This led to the establishment of protective and curative serum therapy, which is based on the power of antitoxin to neutralize the corresponding toxin.

It was now hoped that specific antitoxins could be obtained for all infectious diseases by repeating in each case the scheme I have outlined, but this expectation met with disappointment because only few microbes produce diseases by the secretion of typical toxins, and to this day we have only two potent antitoxic serums for human diseases, namely, antidiptheria serum and antitetanus serum. It is true, however, that many other toxins have been found besides diphtheria and tetanus toxins, and that both in animals and plants. The immunity which is dependent on the neutralization of toxins by specific antitoxins is called *antitoxic immunity* to distinguish it from other forms in whch the mechanism is different and in which it concerns other antibodies than antitoxins.

It is not necessary at this time to speak of the immeasurable benefits of antidiphtheria serum, but a few words may be in order in regard to the use of antitetanus serum, which is of special interest to the surgeon, especially as a preventive of tetanus.

The evidence in favor of the prophylactic value of antitetanic serum is substantial and convincing. I would point first to the success of veterinarians in preventing by means of serum the development of tetanus in horses which are very susceptible to this infection through wounds of various kinds. This practice originated with Nocard. In a district in Paris in which tetanus was prevalent and caused great loss, he stopped its occurrence practically completely by the early use of the serum. In the next place the preventive effectiveness of the serum in the case of human beings would seem to be illustrated in a striking manner by the tetanus epidemic in the maternity wards in Prague. Tetanus developed in the lying-in in spite of everything that could be done until von Rosthorn began to inject antitetanic serum when no further cases developed. Finally it is most significant that when tetanus serum is used as a routine measure in the treatment of wounds contaminated with dirt and other foreign substances, the number of cases of tetanus diminishes noticeably. This is the case among soldiers, both in war and peace, and in large, general surgical clinics. In a group of Paris hospitals there developed eleven cases in seven years and all these cases occurred in persons that by some mischance failed to receive the customary injection of antitetanus serum which is given by routine in all street accidents. According to the reports secured by the Journal of the American Medical Association, the injection of 1500 units of antitoxin in Fourth of July injuries together with thorough opening and cleansing of the wound is proving very generally successful in preventing tetanus. On the basis of the accumulated experience, Kocher would hold any surgeon blamable who does not inject tetanus antitoxin as early as possible in all cases of wounds contaminated with street-dirt. During the last five years there occurred, according to the statistics of the coroner's office, in Chicago, 157 deaths from tetanus in persons wounded in various ways but exclusive of railroad accidents of all kinds; by far the larger number developed in cases of contused and lacerated wounds and of nail punctures of the feet; more cases occur in the summer months than at other times of the year. It is altogether proper to venture the statement that many of these deaths were preventable because tetanus antitoxin is not used regularly. For preventive purposes at least 1500 units of antitoxin should be injected subcutaneously, and repeated in eight days. In infected wounds it is especially desirable that the injections should be repeated. Dusting the wound with dried antitetanus serum is also recommended. While the curative results obtained from antitetanus serum are not so satisfactory as the preventive, to neglect its use would be wholly unwarranted as more cases recover with than without serum. Large doses injected intravenously would seem to offer the best results.

### Anti-Infectious Immunity.

I indicated that in the course of the investigation set on foot by Behring's discovery, it was found that most pathogenic germs do not secrete poisonous substances with antigenic powers, that is, true toxins, as is the case with the bacilli of diphtheria and of tetanus. It was learned, however, from the work of Pfeiffer and others that on injection of certain germs, notably those of typhoid fever and cholera, which appear to cause toxic effects as they are broken up, animals produce bodies that attack the germs themselves and in conjunction with the complement of the blood cause their disintegration or lysis. These bodies are called lysins, lytic amboceptors, or immune bodies; they have specific affinities for the antigens that incite their formation and their function in lysis is to unite the complement with the cell, being in themselves without direct destructive power. Here then we have an antibody which is different from antitoxin in its structure and in its mode of action, and bodies of this kind are produced not only by the injection of bacterial cells but also of animal cells, especially red corpuscles, which have served as a very useful means of investigation into the nature of the immune bodies or amboceptors, and of complement. Amboceptors are quite stble bodies whereas complement, which is not increased by immunization, that is is not produced anew as antibodies are, is a sensitive substance, destroyed by heat at 56° C. for 30 minutes, and endowed with digestive properties which it can exercise, however, only after it is joined to the cell or substance attacked by means of a specific amboceptor. As I shall point out shortly, the phenomena of complement deviation or fixation as well as of allergy or anaphylaxis also depend on amboceptor-complement action, and the full opsonic action of blood serum seems to be owing to a similar mechanism.

Metchnikoff established the general occurrence of phagocytosis in health and disease; Denys and others showed that the fluids of the body are necessary for phagocytosis as they make bacteria susceptible to phagocytic action; and we now know that this property of the body fluids is due to definite substances of the nature of antibodies, and called opsonins by Wright, which attach themselves to microbes and other cells and so alter them that they are taken up by the phagocytes. In some cases it is easy to demonstrate by laboratory methods that bacteria so ingested, e. g. streptococci, pneumococci, anthrax bacilli, are destroyed within the phagocytes whereas they grow freely in suspensions of phagocytes without the presence of opsonin, and in opsonic serum alone. The recent investigations of the opsonins have served to establish more clearly and firmly the great importance of phagocytosis in many infections notably with streptococci, pneumococci, tubercle baccilli, and other bacteria that are not destroyed readily by lysis, and that even in the case of bacteria subject to lysis phagocytosis plays a larger role than first believed. At first opsonins were regarded as simple bodies, but it was soon found that here also it involved two substances, one easily destroyed by heat and not increased by means of antigens, the other relatively resistant to heat, provided with specific affinities, and produced anew in immunization. The heat-resistant opsonic elements are capable of opsonic action by themselves and unite firmly with the objects acted on, but the opsonic effect is increased very much by means of the thermolabile, complement-like element. Whether opsonification is caused by distinct, independent substances or by antibodies with other actions as well, such as lysins, has been discussed a good deal, but we cannot stop to consider this question now.

On injection or introduction, as in natural infection, of certain bacteria, like typhoid and allied bacilli, gonococci, maningococci, and others, the lytic as well as the opsonic powers of the blood and other fluids are increased in a specific manner by the new production of antibodies; in other cases, as with streptococci and pneumococci, it is the opsonins that are increased so far as we can tell now. Because the actions of the lysins and opsonins are directed against microbes themselves rather than against their secretory products, the immunity in the infections in which the new production of these antibodies is an outstanding feature is designated as *anti-infectious* or *antibacterial* to distinguish it from the antitotoxic. Unforunately the immune serums of the anti-infectious type have not proved to be uniformly potent in curative and protective action. The most successful is the antimeningitis serum which appears to destroy meningococci directly as well as to cause increased phagocytosis and endophabocytic destruction and to destroy poisonous substances. Antigonococcus serum seems to act in a similar manner whereas antistreptococcus serum is more exclusively opsonic in its effects. Good effects have been obtained from the use of antigonococcus serum in various subacute and chronic localizations of the gonococcus and it seems not unlikely that even better results and prompter might be achievable if the serum were concentrated and injected in large quantities. The intraspinal injection of antimeningitis serum represents an ideal way of introducing antibodies because it brings them at once in contact with the substances we wish to destroy and neutralize.

And now a brief reference to antistreptococcus serum may be ventured. The chief antibody in this serum is opsonin. Soon after it is drawn, the serum, which is obtained from horses properly injected with streptococci, loses the larger part of its opsonic power as determined outside the body. This loss, however, is due to the disappearance of the heat-sensitive thermolabile, complement-like opsonic element, and fortunately the full power is restored by means of fresh serum of various kinds, including human blood from normal individuals as well as from patients with streptococcus infections. Antistreptococcus serum protects guinea-pigs against virulent streptococci just so long as it is reactivable in this way. It seems then that the potency of antistreptococcus serum depends in large measure on the presence of stable opsonic antibodies which there is good reason to believe are activated in the body. In animals (guinea-pigs, rabbits) as well as in human beings there is an increase of opsonin for streptococci in the blood after injection of anti-streptococcus serum, the amount of increase depending of course on the strength of the serum and on the quantity introduced as well as to some degree on the place of injection, which furthermore greatly influences the time when the relative maximum concentration is established. As in the case of the other antibodies absorption is slowest after subcutaneous injection and the relative concentration in the blood the lowest; on intramuscular injection the absorption is more rapid and the concentration higher: while on intravascular injection the maximum concentration is established at once and apparently with the least loss of opsonin. An as yet unexplained but apparently constant result of injections of antistreptococcus serum of much interest is increase in the phagocytic power of the leukocytes of the blood. There consequently is no question but that antistreptococcus serum has protective and curative powers in proportion, we may assume, roughly speaking, to its opsonic content. In severe cases of erysipelas and of septic fever, Weaver and Tunnicliff of the Memorial Institute find intramuscular injections of good serum, 50 cc. at a time, are followed by rapid clinical improvement coincident with a marked increase in the streptococcus opsonins in the blood and in the phagocytic powers of the leukocytes. In view of these results, it becomes highly desirable that the value of antistreptococcus serum in other severe streptococcus infections, and especially those of puerperal origin, should be studied carefully, and if possible by the statist method.

In view of the fact that we lack standards of strength for antistreptococcus serum, the dosage must be arbitrary, and as the serums on the market vary, uniform results can not be expected. Experience teaches that large doses should be used, and if necessary repeated at frequent intervals. Estimations of the opsonic index may prove of value as a guide to the amount and the time of the reinjections as the aim should be to keep the opsonin content of the blood well above normal. In order to secure rapid absorption, intramuscular injections would be preferable to subcutaneous and in very urgent cases intravenous injection should be considered because it secures the maximum concentration of antibodies in the blood at once. In localized streptococcus infections, as in joints, serous cavities, and soft tissues, the serum might be injected with advantage directly into and about the seat of infection.*

Finally I would emphasize that good antistreptococcus serum undoubtedly may be of value as a preventive of streptococcus infection in neglected and complicated obstetrical cases, and when this infection is feared as a consequence of certain surgical operations.

AGGLUTINATION; PRECIPITATION, COMPLEMENT-FIXATION.

At this point mention may be made of certain reactions of immunity which are regarded as of minor importance in overcoming infection and intoxication yet as dependent at least in large part on distinct antibodies, which follow the same general laws as the antibodies already discussed, and which constitute diagnostic means of great importance. I refer to agglutination, precipitation, and complement fixation. While studying lysis of typhoid bacilli, Gruber and Durham recognized the occurrence of

*It is possible to concentrate the opsonin in antistreptococcus serum by the same methods as are used to concentrate antidiphtheric serum; this may prove to be of practical value in so far as it would reduce the quantity injected; it is possible that on subcutaneous and intramuscular injection the absorption of the opsonin in concentrated serum may be delayed by the relative increase of the protein content.

specific agglutination which almost immediately was applied to the diagnosis of typhoid fever and soon became established as a valuable diagnostic method which now is used everywhere. Agglutinins with different affinities are present in small quantities normally in the blood of man and animals and new production of specific agglutinins occurs with readiness on the introduction of bacteria and other antigenic substances notably red corpuscles. Much use is made of agglutination in solving questions of relationship of bacteria among themselves as well as to infectious processes.

There is a large number of protein substances of bacterial, vegetable, and animal origin which induce the formation of so-called precipitins, the action of which manifests itself in a precipitate when the serum of the injected animal is mixed under proper conditions with a clear solution of the protein injected or some closely related protein. This reaction is of great practical value in forensic blood diagnosis and in the detection of adulteration of meat food products, but at present it is not used as a routine procedure in clinical diagnosis. It is interesting to note that recently Ascoli has developed a precipitin-test for anthrax which is of great value from a veterinary point of view. Extracts of infected organs, especially the spleen, as well as of the bacilli themselves, give an immediate precipitate on coming in contact with anti-anthrax serum, and the active substance in such extracts is very resistant to destructive influences including boiling and decomposition.

The reaction known as complement fixation depends on the fact, first demonstrated by Bordet and Gengou, that when antigen and antibody unite they bind complement. Hence, when fluids containing antigen, the corresponding antibody, and free complement are mixed under proper conditions, the complement soon disappears so that no laking occurs when red corpuscles and the proper lytic amboceptor are added to the mixture. By this method it becomes possible to test as to antigen or antibody as the absence of either leaves the complement free so that laking results. As you know, Wassermann's reaction for syphilis, one of the most important and interesting methods of diagnosis, rests on the assumption that it is possible by typic fixation to demonstrate the absence or presence of specific anti-syphilitic bodies in the serum and cerebrospinal fluid. The method has proved useful also in other diseases and in the study of antibodies in certain therapeutic serums. Even very small quantities of glanders antibodies are detected by this test and its routine use in equine glanders is strongly urged by Mohler and others; undoubtedly the method will prove useful in the diagnosis of human glanders also.

Excepting allergy, which I shall discuss later, we have now considered the principal ways in which the actions of antibodies may manifest themselves, and before proceeding further in this direction it will be well to call to mind the chief facts that are known concerning the formation of antibodies in disease and under experimental conditions.

